# A study on combination of daptomycin with selected antimicrobial agents: in vitro synergistic effect of MIC value of 1 mg/L against MRSA strains

**DOI:** 10.1186/s40360-019-0305-y

**Published:** 2019-05-06

**Authors:** Yi-Chien Lee, Pao-Yu Chen, Jann-Tay Wang, Shan-Chwen Chang

**Affiliations:** 1 Department of Internal Medicine, Fu Jen Catholic University Hospital, Fu Jen Catholic University, New Taipei City, Taiwan; 2 School of Medicine, College of Medicine, Fu Jen Catholic University, New Taipei City, Taiwan; 3 Department of Internal Medicine, National Taiwan University Hospital, 7 Chung-Shan South Road, 100 Taipei, Taiwan; 4 Institute of Infectious Diseases and Vaccinology, National Health Research Institutes, Tsu-Nan County, Taiwan

**Keywords:** Combination therapy, Daptomycin, MRSA, Checkerboard assays

## Abstract

**Background:**

Daptomycin is an important drug used in the treatment of methicillin-resistant *Staphylococcus aureus* (MRSA) infection. A high dose of daptomycin is indicated for an MRSA infection with a minimum inhibitory concentration (MIC) of 1 mg/L for daptomycin. Combination therapies with daptomycin and other antimicrobial agents, including fosfomycin, display in vitro synergism potentially. This study was conducted to investigate the in vitro synergistic effect of daptomycin-based combination therapy against MRSA strains with high daptomycin MIC.

**Method:**

The synergistic effects of daptomycin in combination with fosfomycin, gentamicin, linezolid, oxacillin, or rifampicin against MRSA with an MIC of 1 mg/L for daptomycin were measured using the microbroth checkerboard assay in vitro.

**Result:**

A total of 100 MRSA isolates was tested. The synergistic interactions of the drugs were evaluated using the fractional inhibitory concentration index. The MIC values revealed that all isolates (100%) were found to be susceptible to linezolid, 85% to fosfomycin, 8% to gentamicin, 69% to rifampicin, and no isolate was susceptible to oxacillin. The in vitro synergism rates of daptomycin in combination with fosfomycin, oxacillin, gentamicin, linezolid, and rifampicin were 37, 11, 5, 3, and 1%, respectively.

**Conclusion:**

The combination of daptomycin plus fosfomycin may be an effective therapeutic option for MRSA infection.

**Electronic supplementary material:**

The online version of this article (10.1186/s40360-019-0305-y) contains supplementary material, which is available to authorized users.

## Background

For the past few decades, methicillin-resistant *Staphylococcus aureus* (MRSA) continued to be a major human pathogen causing various dangerous infections, such as bacteremia, endocarditis, and abscess, in both community and hospital (nosocomial infection, health-care associated infection or hospital associated infection) settings [1]. The definition of MRSA nosocomial infection was classified “hospital onset”, if culture confirmed to be MRSA isolates was obtained after hospital day 3 with admission being day 1 [2]. In the United States, MRSA contributes to 25.8% of all *S. aureus* infections and it is associated with approximately 11,000 deaths annually [2]. Its prevalence is particularly higher in Taiwan, as ever more than 80% in intensive care units [3]. Alarmingly, the emergence of multi-drug resistant *S. aureus* has created an international issue that needs to be solved through new treatment options [4]. Based on the treatment guidelines for MRSA infection, vancomycin remained the mainstay of parenteral therapy. However, daptomycin was considered at least as effective as vancomycin in treating MRSA bacteremia, and high-dose daptomycin in combination with another agent, including gentamicin, rifampicin, linezolid, was suggested to be the option against persistent MRSA bacteremia with vancomycin treatment failures [5]. Hence, combinations of various therapeutic strategies may be effective in improving the clinical outcome of patients with severe staphylococcal diseases [6, 7]. Broadly, the rationale of using combination therapy is as follows: provision of wide-spectrum benefits, acquisition of the synergistic effect, and low risk of emergence of drug-resistant strains [8].

Daptomycin is a cyclic lipopeptide antimicrobial agent produced by *Streptomyces roseosporus.* This agent targets the bacterial cell membrane via a calcium-dependent pathway, disrupting electrical potential, altering cell membrane permeability, opening ion channels, and eventually causing cell death [9]. This drug is FDA-approved for adult patients with *S. aureus* infection, including MRSA infection, bloodstream infections, right-sided infective endocarditis, complicated skin and soft tissue infections [5]. Daptomycin kills MRSA more rapidly than glycopeptides. Several studies have reported that daptomycin exhibited a high efficacy against bacteremia caused by MRSA with a high minimum inhibitory concentration (MIC) for vancomycin [10]. Generally, daptomycin at 6 mg/kg/day is recommended for bacteremia cause by MRSA [9]; however, several studies have shown that up to 8 mg/kg/day daptomycin is required for patients with difficult-to-treat MRSA infection, including that caused by MRSA with an MIC of 1 mg/L for daptomycin [11, 12]. In such a clinical scenario, co-administration of daptomycin and other antimicrobial agents with synergistic interaction may be an effective therapeutic strategy.

Several in vitro studies using checkerboard methods have shown that combination therapies with daptomycin and other antimicrobials exhibit various degrees of synergism [13–15]. Moreover, there is limited information on whether similar synergistic effects are successful against MRSA with a high daptomycin MIC. Hence, in the present study, we aimed to determine the synergistic effects of therapy with daptomycin in combination with fosfomycin, gentamicin, linezolid, oxacillin, or rifampicin by measuring the in vitro antibacterial effects of these combination therapies against MRSA strains with a daptomycin MIC of 1 mg/L using the checkerboard method.

## Methods

### Bacterial isolates and identification

A total of 1353 MRSA specimens from sterile sites, including blood, cerebrospinal fluid, ascites, and pleural effusion, were isolated consecutively between January 2012 and December 2015 from 14 participating hospitals. These hospitals included 12 medical centers and 2 regional hospitals located in northern (6 hospitals), central (3 hospitals), southern (4 hospitals), and eastern (1 hospital) Taiwan. The present numbers of bacterial isolates enrolled each year was 300. When the total number of collected MRSA isolates reached 300 in each year, the participating hospital would be informed to stop submitting further samples for that year. Duplicate isolates were excluded. All these strains were identified as *S. aureus* by performing gram staining, catalase-activity test, and coagulase latex agglutination test (automated VITEK-2 system, Biomerieus, France). Methicillin resistance of the cultured *S. aureus* was determined using agar disk diffusion (Kirby-Bauer), according to the guidelines established by the Clinical and Laboratory Standards Institute (CLSI) [16]. These MRSA isolates were sent to the central laboratory of the National Taiwan University Hospital for preservation, until use in the subsequent microbiological studies. The study was approved by the Ethical Committee of the National Taiwan University Hospital (NTUH-IRB No. 201110043RD).

### Antimicrobials and measurement of MIC

The tested antimicrobials were daptomycin (Cubist, Pharmaceuticals, Lexington, MA), fosfomycin (Sigma-Aldrich, St. Louis, USA), gentamicin (Sigma-Aldrich), linezolid (Pfizer, New York, USA), oxacillin (Sigma-Aldrich), and rifampicin (USP; Twinbrook Parkway, Rockville, MD, USA), and they were prepared according to the manufacturers’ instructions. MIC values were determined by broth microdilution in cation-adjusted Mueller-Hinton broth with an inoculum of 5 × 10^5^ CFU/ml in the wells of the microplates. The results were interpreted according to the CLSI guidelines [16]. MIC values represent the lowest drug concentration at which complete inhibition of visible microbial growth is observed. Calcium was added to the growth media containing daptomycin to a final concentration of 50 μg/mL, whereas 25 μg/mL glucose-6-phosphate was added to the medium containing fosfomycin [16]. One hundred MRSA isolates were randomly selected from those with MIC of 1 mg/L to daptomycin using a random digital table for the subsequent experiments. The MIC_50_ and MIC_90_ of and the susceptibilities to each tested drug except daptomycin were calculated.

### Synergy testing using the checkerboard assay

We used the 2-dimensional microbroth checkerboard method to assess the in vitro effects of combinations of various antimicrobial agents against MRSA strains [17]. The tested antimicrobials were daptomycin in combination with fosfomycin, gentamicin, linezolid, oxacillin, or rifampicin. Each antimicrobial agent in the combinations was four dilutions above and four dilutions below the MIC of every drug. The first antimicrobial agent in the combination was serially diluted 2-fold along the ordinate, whereas the second was diluted along the abscissa. Each well of the microtiter plate contained an inoculum of 5 × 10^5^ CFU/mL that had been incubated for 24 h at 35 °C. The MIC of every antimicrobial agent alone and in combination represented the lowest dilution that completely inhibited the growth of the bacterium. The interaction of the drugs in a combination was expressed quantitatively as a fractional inhibitory concentration (FIC) index (FICI) and calculated for each drug combination using the following equation: FICI = FIC_A_ + FIC_B,_ where FIC_A_ = MIC of drug A in a combination/MIC of drug A alone, and FIC_B_ = MIC of drug B in a combination/MIC of drug B alone. The FICI results were interpreted as synergistic (≤0.5), additive (> 0.5 to ≤1), or indifferent (> 1) [17]. FICI results were expressed as percentage.

## Results

### MIC values, susceptibility rates, and FICI results of the antimicrobials

There were 144 MRSA isolates with MIC of 1 mg/L for daptomycin among the 1353 MRSA isolates. For the 100 randomly selected clinical isolates of MRSA with MIC of 1 mg/L for daptomycin, 57 were from six hospitals in northern Taiwan, 14 from three hospitals in central Taiwan, 25 from four hospitals in southern Taiwan, and 4 from one hospital in eastern Taiwan.

The MIC ranges, MIC_50_s, MIC_90_s, and the susceptible rates of the tested antimicrobials for the MRSA strains are listed in Table 1 and the detailed MICs are shown in Additional file 1. All strains (100%) were susceptible to linezolid, 85% to fosfomycin, 8% to gentamicin, 69% to rifampicin, and no strain was susceptible to oxacillin. The MIC_50_, MIC_90_, and MIC_range_ values of the antimicrobials are provided in Table 1.

**Table 1:** MIC values of antimicrobial agents for 100 MRSA strains (daptomycin MIC = 1) and susceptibility rates

	MIC values (μg/mL)			Susceptibility (%)
	MIC_50_	MIC_90_	MIC_range_	
Fosfomycin	4	> 128	< 0.5 - > 128	85
Gentamycin	> 256	> 256	0.125 - > 256	8
Linezolid	2	2	0.5–4	100
Oxacillin	> 256	> 256	8 - > 256	0
Rifampicin	0.008	32	< 0.004 - > 64	69

**Fig. 1:**
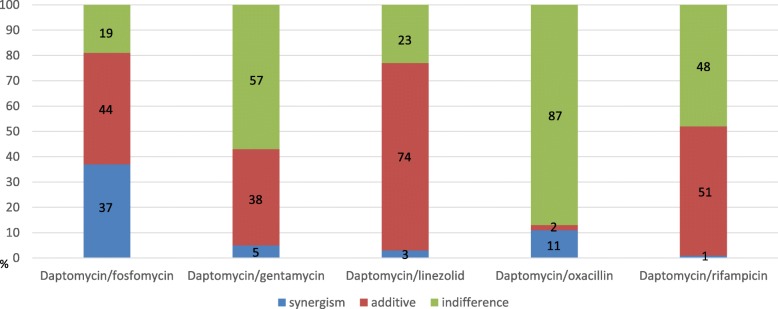
Daptomycin combined with different kinds of antimicrobial agents demonstrated various results of the in vitro antibacterial effects

The FICI values of the combination therapies against the MRSA strains are listed in Table 2 and the in vitro antibacterial effects are demonstrated in Fig. 1. The highest synergistic effect (FICI: ≤0.5) was observed for daptomycin in combination with fosfomycin (37%), which was significantly higher than that in the combinations with oxacillin (11%, *p* < 0.0001), that with gentamicin (5%, *p* < 0.0001), that with linezolid (3%, *p* < 0.0001), and that with rifampicin (1%, *p* < 0.0001). In addition, the additive effect (FICI: > 0.5 to ≤1) in increasing order was 2% for oxacillin, 38% for gentamicin, 44% for fosfomycin, 51% for rifampicin, and 74% for linezolid. The combination of daptomycin and fosfomycin exhibited the highest synergistic/additive effect (81%) against the MRSA strains. A majority (87%) of indifference effect (FICI: > 1) was observed for the daptomycin and oxacillin combination.

**Table 2:** Interpreted FICI results of antimicrobial agents for 100 MRSA strains (daptomycin MIC = 1)

Combination	Interpreted FICI results, (%)		
	synergism	additive	indifference
Daptomycin/fosfomycin	37	44	19
Daptomycin/gentamycin	5	38	57
Daptomycin/linezolid	3	74	23
Daptomycin/oxacillin	11	2	87
Daptomycin/rifampicin	1	51	48

## Discussion

The development of optimal therapeutic strategies for serious MRSA infections remains of great interest because glycopeptide monotherapy has multiple disadvantages, including varied tissue penetration, slow bacterial killing, and emergence of drug-resistant strains [6]. Daptomycin, which has a rapid bactericidal effect against MRSA, may be an ideal treatment option [11]; however, it needs to be administered at high doses [12] and has poor prognosis [18] for patients with high MIC for daptomycin. Thus, combination therapy with daptomycin might overcome the aforementioned disadvantages. To our knowledge, this is the first in vitro study that evaluated the potential effects of a combination therapy with daptomycin and other antimicrobial agents against MRSA strains with MIC of 1 mg/L for daptomycin. In the present study, combination therapy with daptomycin and fosfomycin had the highest synergistic/additive effects against the 100 MRSA strains.

Fosfomycin, a phosphonic acid derivative first identified in 1969 [19], has a good antibacterial effect against various drug-resistant gram-positive cocci, including MRSA. In clinical settings, it is usually combined with other antimicrobials because an in vitro study has reported the rapid development of resistance to this drug [20]. Several studies have shown the synergistic effects of combination therapy with daptomycin and fosfomycin in vitro and in vivo [21, 22]. Despite limited clinical experience, several patients with MRSA bacteremia have shown successful outcomes via such a therapy [23]. In addition, our findings were supported by those of a similar study, which reported that the in vitro synergistic effect of daptomycin combined with fosfomycin is 100% [15]. The difference in synergistic effects between the previous and present studies may be due to the differences in susceptibility to fosfomycin and MIC for daptomycin. The exact mechanism underlying the synergism is still unclear. However, fosfomycin has a unique inhibitory effect on the early stage of peptidoglycan synthesis by inhibiting the formation of N-acetylmuramic acid, which may increase daptomycin binding because of alteration in the electrical charge of the outer bacterial membrane [24]. Additionally, daptomycin combined with fosfomycin could preserve daptomycin susceptibility against MRSA strains with high MIC, which made maintenance of prior therapeutic dose of daptomycin for MRSA possible clinically. However, further studies need to be conducted to confirm the above-mentioned hypothesis.

Linezolid, the first marketed synthetic oxazolidinone drug, has an inhibitory effect on protein synthesis by affecting the 50S subunit of the bacterial ribosome [25]. It is an FDA-approved drug for skin and soft tissue infection and nosocomial pneumonia caused by MRSA, although the bacteriostatic entity may preclude its clinical use [5]. A study involving the addition of carbapenem to the parent linezolid showed a prominent synergistic effect against MRSA infection [26]. Moreover, the effects of therapy with daptomycin in combination with linezolid have been investigated. Parra-Ruiz et al. [27] reported that a daptomycin/linezolid combination is more effective than a single drug in an in vitro biofilm model. Similar results were observed using a model of simulated endocardial vegetations [28]. However, indifferent and antagonistic interactions between drugs in a combination were shown by Kelesidis et al. [29], whose results were comparable to our findings: a small proportion (3%) of synergism and large proportion (74%) of additive effects. Hence, further studies need to be performed to elucidate the efficacy of daptomycin/linezolid combination against MRSA.

A synergistic effect between beta-lactams and daptomycin against MRSA strains was observed in a previous in vitro study [6]. Several in vitro [13, 30] and animal studies [31], have shown that daptomycin/oxacillin combination has a synergistic effect on most tested strains, and the addition of oxacillin to daptomycin in a case series led to the rapid eradication of MRSA bacteremia [32]. The following are proposed mechanisms that may explain these synergistic effects: a decline in the surface charge of MRSA by oxacillin increases daptomycin membrane binding, death of bacteria [32], and activity of oxacillin-mediated innate host defense peptides against MRSA [33]. However, currently, beta-lactam combination therapy is not suggested in the Infectious Diseases Society of America guidelines [5]. In our study, only 11% of the tested MRSA strains were consistent with the synergistic findings, and other strains with indifferent results may have to be further examined in an in vitro study to elucidate this difference.

Co-administration of daptomycin and gentamicin had potential synergistic activities against *S. aureus* in time-killing analyses, as verified by Debbia et al. [21]. Nevertheless, several in vitro and in vivo studies have reported varied results: some reported synergy [13, 34], whereas others did not [35, 36]. Synergistic interaction of up to 68% was reported by Aktas et al. [15], which contradicts the 5% synergism observed in our study. This distinction may be due to the differences in the bacterial strains used. A previous random control clinical trial for evaluating the efficacy of daptomycin with and without gentamicin against *S. aureus* infective endocarditis was terminated prematurely after only 24 patients were recruited [6], making the effects of this combination even more inconclusive.

The use of rifampicin as a single agent against MRSA infection is associated with the rapid development of drug resistance [37], and the effectiveness of the rifampicin and daptomycin combination for increasing the clearance of MRSA biofilm has been reported previously [38]. Studies using animal models and retrospective reviews [39, 40] and case reports [41] have also confirmed the synergy and clinical success of daptomycin in combination with rifampicin. Rose et al. [14] showed via checkerboard analysis that the in vitro synergy of this combination was 75%, with 100% predictive value for treatment outcome. However, a similar study [19] showed poor synergism (12%), which is comparable to that observed in our study (1%). Therefore, owing to incomplete clinical data, as no clinical outcome associated with daptomycin combined with rifampicin is demonstrated in our study and probably different epidemiological background of bacterial isolates between our present study and previous ones, the benefits of this combination therapy are unclear.

Our study had certain limitations. First, although the present study showed the synergistic effects of daptomycin in combination with some drugs, the insufficient clinical data limited our interpretation of the correlation between the in vitro studies and treatment outcome. Second, this study was conducted using the population of a single country, Taiwan; thus worldwide generalization should be made carefully. Finally, some antimicrobial agents effectively against MRSA, like ceftaroline (not available in Taiwan when this manuscript was written), were not included for assessment of the in vitro antibacterial effects.

## Conclusions

In conclusion, our study showed that daptomycin in combination with fosfomycin had a high synergistic/additive effect against MRSA with an MIC of 1 mg/L for daptomycin. Hence, combination therapy with daptomycin and fosfomycin may be an effective therapeutic strategy for MRSA infection with high MIC for daptomycin as compared to monotherapy. However, further clinical research should be conducted to verify the synergistic mechanism of these antimicrobial agents.

## Additional file:

Additional file 1: The MIC of the five antimicrobial agents to those 100 MRSA strains. (PDF 283 kb)

## Abbreviations

CFU: Colony-forming unit
CLSI: Clinical and Laboratory Standards Institute
FDA: Food and Drug Administration
FICI: Fractional inhibitory concentration index
MIC: Minimum inhibitory concentration
MRSA: Methicillin resistant *Staphylococcus aureus*
