# Supplementary material for: A study on combination of daptomycin with selected antimicrobial agents: in vitro synergistic effect of MIC value of 1 mg/L against MRSA strains
Source: BMC Pharmacol Toxicol. 2019 May 6;20:25. doi: 10.1186/s40360-019-0305-y (PMC6503441; doi:10.1186/s40360-019-0305-y)
Supplement: Supplementary file 1 — The MIC of the five antimicrobial agents to those 100 MRSA strains. (PDF 283 kb) [file 40360_2019_305_MOESM1_ESM.pdf]

Supplementary file

	Antimicrobial agents				
MIC / No.	Fosfomycin	Gentamicin	Linezolid	Oxacillin	Rifampicin
1	4	>256	2	>256	>64
2	2	>256	2	>256	0.008
3	4	>256	4	>256	>64
4	8	>256	4	>256	0.008
5	1	>256	2	>256	0.008
6	>128	128	4	>256	2
7	16	256	4	>256	0.008
8	8	>256	4	>256	0.008
9	2	256	4	>256	0.008
10	1	>256	2	>256	0.008
11	8	256	2	>256	<0.004
12	16	>256	2	>256	2
13	8	>256	4	>256	2
14	2	>256	4	>256	0.008
15	4	>256	4	>256	0.008
16	8	256	2	>256	0.008
17	4	128	2	>256	>64
18	2	>256	2	>256	0.008
19	2	256	2	>256	1
20	2	>256	2	>256	0.008
21	1	128	2	>256	0.008
22	2	>256	1	>256	0.008
23	2	>256	1	>256	0.008
24	2	>256	2	>256	0.008
25	2	>256	2	>256	0.008
26	>128	128	1	>256	>64
27	8	>256	1	>256	0.008
28	2	>256	1	>256	0.008
29	>128	128	1	>256	2
30	8	>256	2	>256	0.008
31	4	128	1	>256	0.008
32	8	>256	2	>256	0.06
33	4	>256	2	>256	0.008

34	4	>256	2	>256	>1
35	4	>256	2	>256	0.008
36	2	256	1	>256	0.008
37	4	>256	2	>256	0.008
38	128	256	2	>256	2
39	2	>256	2	>256	0.008
40	2	256	2	>256	0.008
41	4	256	2	>256	0.008
42	2	>256	2	>256	0.008
43	128	256	1	>256	>64
44	2	128	2	>256	0.008
45	2	0.25	2	128	0.015
46	16	256	2	256	2
47	>128	128	1	>256	1
48	2	>256	1	>256	0.015
49	4	256	1	>256	0.008
50	4	>256	1	>256	<0.004
51	8	>256	2	>256	0.008
52	4	128	1	>256	>1
53	4	256	2	>256	1
54	>128	128	1	>256	1
55	2	>256	1	>256	0.008
56	>128	256	4	>256	1
57	2	>256	1	>256	0.008
58	>128	128	1	>256	32
59	1	>256	1	>256	32
60	<0.5	>256	0.5	>256	>64
61	32	>256	1	>256	2
62	<0.5	32	2	256	0.015
63	2	>256	1	>256	>64
64	2	256	2	>256	>64
65	2	128	2	32	0.008
66	2	32	1	256	0.015
67	4	>256	1	>256	>64
68	2	>256	1	>256	0.008
69	2	>256	2	>256	0.008
70	4	>256	2	>256	<0.004

71	2	8	2	>256	0.008
72	2	>256	1	256	0.008
73	4	>256	1	>256	0.008
74	4	64	2	32	4
75	>128	128	2	>256	2
76	8	>256	1	>256	0.008
77	8	128	1	>256	0.008
78	2	0.25	1	>256	0.008
79	<0.5	0.125	2	8	0.008
80	2	0.25	2	>256	0.008
81	4	>256	2	>256	>64
82	>128	128	1	>256	2
83	16	>256	2	>256	0.008
84	2	4	2	128	0.015
85	>128	128	1	>256	2
86	>128	128	1	>256	2
87	2	0.25	2	128	0.008
88	>128	128	2	>256	2
89	4	>256	2	>256	0.008
90	1	>256	1	128	0.008
91	4	0.25	2	128	0.015
92	16	<1	2	128	0.008
93	2	>256	1	>256	>1
94	4	>256	2	>256	2
95	2	>256	2	>256	1
96	2	>256	2	>256	1
97	2	>256	2	>256	2
98	4	>256	2	>256	2
99	2	>256	2	>256	1
100	>128	64	1	>256	0.008
